# Bis{2-[1-(8-hydroxy-2-quinolylmethyl)-1*H*-benzimidazol-2-yl]quinolin-8-ol} toluene solvate

**DOI:** 10.1107/S1600536810004794

**Published:** 2010-02-20

**Authors:** Hui-Li Chen, Qi Ma, Qing-Ming Wang

**Affiliations:** a Institute of Molecular Science, Key Laboratory of Chemical Biology and Molecular, Engineering of the Education Ministry, Shanxi University, Taiyuan, Shanxi 030006, People’s Republic of China; b College of Chemistry and Chemical Engineering , Shanxi Datong University, Datong, Shanxi 037009, People’s Republic of China

## Abstract

Crystals of the title compound, 2C_26_H_18_N_4_O_2_·C_7_H_8_, were obtained from the reaction of 8-hydroxy­quinoline with 1,2-phenyl­enediamine in methanol and recrystallized from toluene. The compound contains three essentially planar ring systems: the benzimidazole ring (r.m.s. deviation = 0.039 Å) and two 8-hydroxy­quinoline rings (r.m.s. deviations of 0.0056 Å in both rings). The benzimidazole ring and one 8-hydroxy­quinoline ring are almost co-planar, forming a dihdral angle of 3.1 (2)°. The other 8-hydroxy­quinoline ring is almost perpendicular to the benzimidazole plane with a dihedral angle of 86.2 (2)°. Intra­molecular O—H⋯N contacts are present. The crystal structure is stabilized by inter­molecular O—H⋯N inter­actions. The complete toluene molecule is generated by crystallographic inversion symmetry; therefore its methyl group is disordered over two sites of equal occupancy.

## Related literature

For the use of the reaction of *o*-phenyl­enediamine with excess aldehyde without an oxidant to produce a Shiff base compound containing two —N=CH— bonds, see: Chen & Martell (1987 ▶); Wang *et al.* (1994 ▶). Similar benzimidazole derivatives have been obtained, see: Dege *et al.* (2006 ▶); Yang *et al.* (2004 ▶). For the preparation of benzimidazole, see: Boufatah *et al.* (2004 ▶); Grimmet (1997 ▶); Kumar *et al.* (1981 ▶); Srivastava & Venkataramair (1988 ▶). 
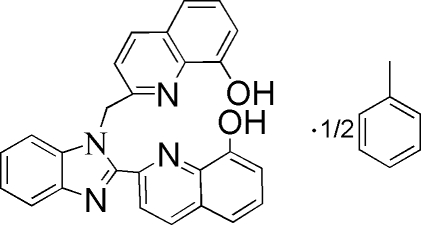


## Experimental

### 

#### Crystal data

2C_26_H_18_N_4_O_2_·C_7_H_8_
*M* *_r_* = 929.02
Triclinic, 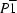

*a* = 8.014 (7) Å
*b* = 12.669 (11) Å
*c* = 12.727 (11) Å
α = 112.979 (10)°
β = 90.881 (11)°
γ = 100.966 (11)°
*V* = 1162.1 (17) Å^3^
*Z* = 1
Mo *K*α radiation
μ = 0.09 mm^−1^
*T* = 295 K
0.30 × 0.20 × 0.15 mm

#### Data collection

Bruker SMART CCD area-detector diffractometer
Absorption correction: multi-scan (*SADABS*; Sheldrick, 2001 ▶) *T*_min_ = 0.975, *T*_max_ = 0.987
6333 measured reflections
4077 independent reflections
3049 reflections with *I* > 2σ(*I*)
*R*_int_ = 0.024

#### Refinement

*R*[*F* ^2^ > 2σ(*F* ^2^)] = 0.051
*wR*(*F* ^2^) = 0.156
*S* = 1.03
4077 reflections
329 parameters
2 restraints
H-atom parameters constrained
Δρ_max_ = 0.40 e Å^−3^
Δρ_min_ = −0.32 e Å^−3^

### 

Data collection: *SMART* (Bruker, 2000 ▶); cell refinement: *SAINT* (Bruker, 2000 ▶); data reduction: *SAINT*; program(s) used to solve structure: *SHELXS97* (Sheldrick, 2008 ▶); program(s) used to refine structure: *SHELXL97* (Sheldrick, 2008 ▶); molecular graphics: *SHELXTL/PC* (Sheldrick, 2008 ▶); software used to prepare material for publication: *SHELXTL/PC*.

## Supplementary Material

Crystal structure: contains datablocks I. DOI: 10.1107/S1600536810004794/bv2138sup1.cif

Structure factors: contains datablocks I. DOI: 10.1107/S1600536810004794/bv2138Isup2.hkl

Additional supplementary materials:  crystallographic information; 3D view; checkCIF report

## Figures and Tables

**Table 1:** Hydrogen-bond geometry (Å, °)

*D*—H⋯*A*	*D*—H	H⋯*A*	*D*⋯*A*	*D*—H⋯*A*
O1—H1⋯N1	0.82	2.29	2.745 (3)	116
O1—H1⋯N4	0.82	2.47	3.131 (3)	139
O2—H2⋯N4	0.82	2.27	2.722 (3)	116
O2—H2⋯N2^i^	0.82	2.55	3.145 (3)	131

Symmetry code: (i) 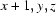.

**Fig. 1.**
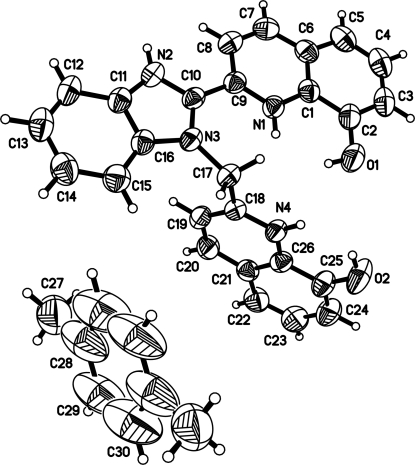
The structure of the title compound,with the atom-numbering scheme.Displacement ellipsoids are drawn at the 50% probability level.

**Fig. 2.**
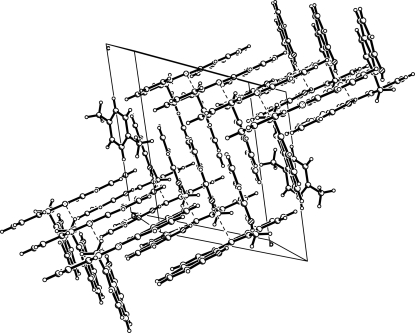
The packing view of the title compound.

## supplementary crystallographic information

### Comment

In most cases, without oxidant, the reaction of *o*-phenylenediamine with excess aldehyde produces a Shiff-base compound containing two —N═CH— bonds (Chen *et al.*,1987; Wang *et al.*,1994). However, in our case, the reaction of *o*-phenylenediamine with 3 equivalents of 8-hydroxyquinoline-2-aldehyde did not form the desired compound. Instead, the reaction produced a novel 2-substituted benzimidazole. Similar benzimidazole derivatives were also obtained by Dege and Yang (Dege *et al.*, 2006; Yang *et al.*, 2004). Usually, one of the general routes for synthesis of benzimidazole involves the reaction of a carboxylic acid with *o*-phenylenediamine in the presence of a strong acid (Grimmet *et al.*, 1997; Boufatah *et al.*, 2004). Another typical procedure involves heating *o*-phenylenediamine with an aldehyde in the presence of oxidant, such as Pb(OAc)_4_ (Kumar *et al.*, 1981), BaMnO_4_ (Srivastava *et al.*, 1988).

The molecular structure and a packing diagram of the title compound are illustrated in Figs 1 and 2, respectively. Selected geometric parameters are listed in Table 1.The compound contains 3 planar rings. One is the benzimidazole ring (N2, N3, C10—C16); the others are the 8-hydroxyquinoline rings. The 8-hydroxyquinoline ring [A(N1,O1,C1—C9)] attached to C10, is almost coplanar with the benzimidazole ring (with a dihedral angle of 3.1 (2)°). The other 8-hydroxyquinoline group [B(N4,O2,C18—C26)], was attached to the C17 methylene group almost perpendicular to the benzimidazole plane (with a dihedral angle of 93.8 (2)°). Two 8-hydroxyquinoline rings (A and B) form a dihedral angle of 96.5 (2)°. The C17—C18,C17—N3 and N2—C10 bond distances are 1.513 (3), 1.462 (3) and 1.327 (3) Å, which are similar to the corresponding bond lengths in 1-(thiophen-2-ylmethyl)-2-(thiophen-2-yl)-1*H*-benzimidazole (1.501 (3), 1.452 (3) and 1.315 (3) Å) (Dege *et al.*, 2006). There is a strong intermolecular between O2—H2···N2(*x* + 1,*y*,*z*), with a H2···N2 distance of 2.55 Å (Figure 2, Table 2).

### Experimental

A solution of 1,2-diaminobenzene (0.001 mol) in absolute methanol (20 ml) was added in small portions to a solution of 8-hydroxyquinoline-2-aldehyde (0.003 mol) in absolute methanol (30 ml). The reaction mixture was maintained at 348 K for 2 h,and was monitored by TLC. The resulting precipitation was washed with methanol, dried and recrystallized from toluene. ^1^H NMR(d6-DMSO):9.46(s,1*H*),9.33(s,1*H*),8.56(d,1*H*),8.47(d,1*H*),8.15(d,1*H*), 7.82–7.85(m,2*H*),7.42–7.48(m,2*H*),7.08–7.40(m,7*H*),6.73(s,2*H*).

### Refinement

Toluene molecule is located at a symmetrical center, so 4-H of toluene is not present. H atoms attached to C atoms were placed in geometrically idealized positions with C*sp*^2^—H = 0.93 Å and C*sp*^3^—H = 0.96 Å, and were constrained to ride on their parent atoms.

### Crystal data

**Table:**

2C_26_H_18_N_4_O_2_·C_7_H_8_	*Z* = 1
*M**_r_* = 929.02	*F*(000) = 486
Triclinic, *P*1	*D*_x_ = 1.327 Mg m^−^^3^
Hall symbol: -P 1	Mo *K*α radiation, λ = 0.71073 Å
*a* = 8.014 (7) Å	Cell parameters from 2466 reflections
*b* = 12.669 (11) Å	θ = 3.0–25.9°
*c* = 12.727 (11) Å	µ = 0.09 mm^−^^1^
α = 112.979 (10)°	*T* = 295 K
β = 90.881 (11)°	Block, yellow
γ = 100.966 (11)°	0.30 × 0.20 × 0.15 mm
*V* = 1162.1 (17) Å^3^	

### Data collection

**Table:**

Bruker SMART CCD area-detector diffractometer	4077 independent reflections
Radiation source: fine-focus sealed tube	3049 reflections with *I* > 2σ(*I*)
graphite	*R*_int_ = 0.024
phi and ω scans	θ_max_ = 25.1°, θ_min_ = 1.8°
Absorption correction: multi-scan (*SADABS*; Sheldrick, 2001)	*h* = −9→7
*T*_min_ = 0.975, *T*_max_ = 0.987	*k* = −15→14
6333 measured reflections	*l* = −12→15

### Refinement

**Table:**

Refinement on *F*^2^	Secondary atom site location: difference Fourier map
Least-squares matrix: full	Hydrogen site location: inferred from neighbouring sites
*R*[*F*^2^ > 2σ(*F*^2^)] = 0.051	H-atom parameters constrained
*wR*(*F*^2^) = 0.156	*w* = 1/[σ^2^(*F*_o_^2^) + (0.0768*P*)^2^ + 0.2872*P*] where *P* = (*F*_o_^2^ + 2*F*_c_^2^)/3
*S* = 1.02	(Δ/σ)_max_ < 0.001
4077 reflections	Δρ_max_ = 0.40 e Å^−^^3^
329 parameters	Δρ_min_ = −0.32 e Å^−^^3^
2 restraints	Extinction correction: *SHELXL*, Fc^*^=kFc[1+0.001xFc^2^λ^3^/sin(2θ)]^-1/4^
Primary atom site location: structure-invariant direct methods	Extinction coefficient: 0.033 (4)

### Special details

**Table:**

Geometry. All e.s.d.'s (except the e.s.d. in the dihedral angle between two l.s. planes) are estimated using the full covariance matrix. The cell e.s.d.'s are taken into account individually in the estimation of e.s.d.'s in distances, angles and torsion angles; correlations between e.s.d.'s in cell parameters are only used when they are defined by crystal symmetry. An approximate (isotropic) treatment of cell e.s.d.'s is used for estimating e.s.d.'s involving l.s. planes.
Refinement. Refinement of *F*^2^ against ALL reflections. The weighted *R*-factor *wR* and goodness of fit *S* are based on *F*^2^, conventional *R*-factors *R* are based on *F*, with *F* set to zero for negative *F*^2^. The threshold expression of *F*^2^ > σ(*F*^2^) is used only for calculating *R*-factors(gt) *etc*. and is not relevant to the choice of reflections for refinement. *R*-factors based on *F*^2^ are statistically about twice as large as those based on *F*, and *R*- factors based on ALL data will be even larger.

### Fractional atomic coordinates and isotropic or equivalent isotropic displacement parameters (Å^2^)

**Table:**

	*x*	*y*	*z*	*U*_iso_*/*U*_eq_	Occ. (<1)
C1	0.5707 (3)	0.12196 (18)	−0.05782 (17)	0.0455 (5)	
C2	0.7346 (3)	0.0952 (2)	−0.0469 (2)	0.0535 (6)	
C3	0.7601 (3)	−0.0148 (2)	−0.1092 (2)	0.0624 (6)	
H3	0.8668	−0.0316	−0.1027	0.075*	
C4	0.6260 (4)	−0.1028 (2)	−0.1830 (2)	0.0676 (7)	
H4	0.6453	−0.1773	−0.2248	0.081*	
C5	0.4679 (3)	−0.0808 (2)	−0.1944 (2)	0.0633 (6)	
H5	0.3807	−0.1402	−0.2435	0.076*	
C6	0.4366 (3)	0.03220 (19)	−0.13169 (18)	0.0509 (5)	
C7	0.2767 (3)	0.0626 (2)	−0.13557 (19)	0.0556 (6)	
H7	0.1833	0.0059	−0.1805	0.067*	
C8	0.2582 (3)	0.17396 (19)	−0.07426 (19)	0.0524 (6)	
H8	0.1532	0.1942	−0.0774	0.063*	
C9	0.4018 (2)	0.25868 (18)	−0.00545 (17)	0.0434 (5)	
C10	0.3807 (2)	0.37994 (17)	0.05742 (16)	0.0421 (5)	
C11	0.2623 (3)	0.52959 (18)	0.12008 (18)	0.0462 (5)	
C12	0.1518 (3)	0.6075 (2)	0.1430 (2)	0.0554 (6)	
H12	0.0394	0.5825	0.1100	0.066*	
C13	0.2159 (3)	0.7227 (2)	0.2162 (2)	0.0625 (6)	
H13	0.1452	0.7763	0.2325	0.075*	
C14	0.3845 (3)	0.7607 (2)	0.2665 (2)	0.0652 (7)	
H14	0.4228	0.8390	0.3160	0.078*	
C15	0.4960 (3)	0.6855 (2)	0.2448 (2)	0.0569 (6)	
H15	0.6081	0.7112	0.2783	0.068*	
C16	0.4318 (2)	0.56921 (18)	0.16998 (17)	0.0441 (5)	
C17	0.6860 (2)	0.47670 (18)	0.15656 (18)	0.0437 (5)	
H17A	0.7497	0.5569	0.1811	0.052*	
H17B	0.7314	0.4293	0.0878	0.052*	
C18	0.7154 (2)	0.43377 (16)	0.24939 (16)	0.0411 (5)	
C19	0.5972 (3)	0.43317 (19)	0.33017 (18)	0.0511 (5)	
H19	0.4935	0.4545	0.3249	0.061*	
C20	0.6374 (3)	0.4007 (2)	0.41619 (19)	0.0557 (6)	
H20	0.5605	0.4004	0.4701	0.067*	
C21	0.7938 (3)	0.36783 (18)	0.42466 (17)	0.0481 (5)	
C22	0.8471 (3)	0.3341 (2)	0.5118 (2)	0.0632 (6)	
H22	0.7776	0.3338	0.5695	0.076*	
C23	1.0006 (4)	0.3022 (2)	0.5104 (2)	0.0702 (7)	
H23	1.0346	0.2799	0.5675	0.084*	
C24	1.1081 (3)	0.3024 (2)	0.4245 (2)	0.0680 (7)	
H24	1.2123	0.2804	0.4252	0.082*	
C25	1.0602 (3)	0.3348 (2)	0.3397 (2)	0.0556 (6)	
C26	0.9023 (2)	0.36878 (17)	0.33817 (17)	0.0440 (5)	
C27	0.6666 (8)	0.9326 (7)	0.5448 (6)	0.109 (2)	0.50
H27A	0.6352	0.8484	0.5116	0.163*	0.50
H27B	0.5904	0.9635	0.5112	0.163*	0.50
H27C	0.6590	0.9610	0.6260	0.163*	0.50
C28	0.8398 (8)	0.9701 (3)	0.5231 (4)	0.1311 (17)	
C29	0.8662 (8)	0.9652 (4)	0.4163 (4)	0.151 (2)	
H29	0.7753	0.9409	0.3601	0.181*	
C30	1.0280 (8)	0.9965 (4)	0.3939 (4)	0.141 (2)	
H30	1.0493	0.9953	0.3219	0.169*	
N1	0.5530 (2)	0.23373 (15)	0.00443 (14)	0.0448 (4)	
N2	0.2332 (2)	0.41096 (15)	0.05066 (15)	0.0486 (5)	
N3	0.5069 (2)	0.47278 (14)	0.12805 (14)	0.0426 (4)	
N4	0.8615 (2)	0.40046 (14)	0.25095 (14)	0.0437 (4)	
O1	0.8628 (2)	0.17832 (16)	0.02683 (16)	0.0740 (5)	
H1	0.8293	0.2394	0.0589	0.111*	
O2	1.1662 (2)	0.3371 (2)	0.25782 (18)	0.0811 (6)	
H2	1.1174	0.3512	0.2093	0.122*	

### Atomic displacement parameters (Å^2^)

**Table:**

	*U*^11^	*U*^22^	*U*^33^	*U*^12^	*U*^13^	*U*^23^
C1	0.0529 (12)	0.0487 (12)	0.0422 (12)	0.0166 (9)	0.0120 (9)	0.0232 (10)
C2	0.0539 (13)	0.0595 (14)	0.0556 (14)	0.0219 (11)	0.0143 (10)	0.0271 (11)
C3	0.0682 (16)	0.0667 (16)	0.0672 (16)	0.0343 (13)	0.0272 (12)	0.0325 (13)
C4	0.091 (2)	0.0566 (15)	0.0625 (16)	0.0302 (14)	0.0287 (14)	0.0244 (12)
C5	0.0789 (17)	0.0514 (14)	0.0552 (14)	0.0112 (12)	0.0127 (12)	0.0176 (11)
C6	0.0610 (14)	0.0511 (13)	0.0430 (12)	0.0117 (10)	0.0097 (10)	0.0214 (10)
C7	0.0552 (13)	0.0541 (14)	0.0526 (13)	0.0027 (10)	−0.0015 (10)	0.0204 (11)
C8	0.0441 (12)	0.0589 (14)	0.0560 (13)	0.0092 (10)	−0.0010 (10)	0.0260 (11)
C9	0.0438 (11)	0.0506 (12)	0.0424 (11)	0.0128 (9)	0.0048 (8)	0.0243 (9)
C10	0.0395 (11)	0.0515 (12)	0.0420 (11)	0.0120 (9)	0.0034 (8)	0.0249 (9)
C11	0.0455 (11)	0.0545 (13)	0.0492 (12)	0.0162 (9)	0.0099 (9)	0.0291 (10)
C12	0.0471 (12)	0.0673 (15)	0.0682 (15)	0.0250 (11)	0.0153 (10)	0.0383 (13)
C13	0.0691 (16)	0.0657 (16)	0.0717 (16)	0.0334 (13)	0.0234 (12)	0.0382 (13)
C14	0.0793 (17)	0.0525 (14)	0.0670 (16)	0.0222 (12)	0.0132 (13)	0.0236 (12)
C15	0.0582 (14)	0.0547 (14)	0.0584 (14)	0.0136 (11)	0.0034 (11)	0.0227 (11)
C16	0.0465 (11)	0.0502 (12)	0.0455 (12)	0.0166 (9)	0.0089 (9)	0.0267 (10)
C17	0.0359 (10)	0.0505 (12)	0.0486 (12)	0.0099 (8)	0.0023 (8)	0.0236 (9)
C18	0.0375 (10)	0.0424 (11)	0.0410 (11)	0.0084 (8)	0.0008 (8)	0.0142 (9)
C19	0.0423 (11)	0.0665 (14)	0.0477 (13)	0.0199 (10)	0.0052 (9)	0.0225 (11)
C20	0.0548 (13)	0.0716 (15)	0.0456 (13)	0.0202 (11)	0.0140 (10)	0.0254 (11)
C21	0.0557 (13)	0.0482 (12)	0.0412 (12)	0.0143 (10)	0.0029 (9)	0.0173 (9)
C22	0.0791 (17)	0.0717 (16)	0.0489 (14)	0.0247 (13)	0.0084 (12)	0.0309 (12)
C23	0.0856 (19)	0.0767 (17)	0.0633 (16)	0.0304 (14)	−0.0019 (13)	0.0381 (14)
C24	0.0625 (15)	0.0780 (17)	0.0804 (18)	0.0291 (13)	0.0023 (13)	0.0431 (14)
C25	0.0489 (13)	0.0634 (14)	0.0669 (15)	0.0196 (11)	0.0063 (11)	0.0357 (12)
C26	0.0437 (11)	0.0418 (11)	0.0466 (12)	0.0084 (9)	−0.0012 (9)	0.0184 (9)
C27	0.126 (6)	0.111 (6)	0.093 (5)	0.052 (5)	0.008 (5)	0.033 (4)
C28	0.243 (5)	0.064 (2)	0.083 (3)	0.045 (3)	−0.029 (3)	0.0222 (18)
C29	0.262 (7)	0.096 (3)	0.094 (3)	0.027 (4)	−0.041 (4)	0.044 (2)
C30	0.268 (7)	0.085 (3)	0.069 (3)	0.036 (4)	−0.027 (4)	0.031 (2)
N1	0.0448 (10)	0.0504 (10)	0.0444 (10)	0.0156 (8)	0.0062 (7)	0.0220 (8)
N2	0.0403 (9)	0.0540 (11)	0.0579 (11)	0.0146 (8)	0.0035 (8)	0.0270 (9)
N3	0.0383 (9)	0.0491 (10)	0.0464 (10)	0.0136 (7)	0.0035 (7)	0.0237 (8)
N4	0.0381 (9)	0.0495 (10)	0.0473 (10)	0.0131 (7)	0.0031 (7)	0.0217 (8)
O1	0.0539 (10)	0.0736 (12)	0.0870 (13)	0.0276 (9)	0.0027 (9)	0.0180 (10)
O2	0.0540 (10)	0.1300 (17)	0.1009 (15)	0.0461 (11)	0.0256 (10)	0.0773 (13)

### Geometric parameters (Å, °)

**Table:**

C1—N1	1.363 (3)	C17—C18	1.513 (3)
C1—C6	1.415 (3)	C17—H17A	0.9700
C1—C2	1.435 (3)	C17—H17B	0.9700
C2—O1	1.354 (3)	C18—N4	1.320 (3)
C2—C3	1.365 (3)	C18—C19	1.410 (3)
C3—C4	1.406 (4)	C19—C20	1.365 (3)
C3—H3	0.9300	C19—H19	0.9300
C4—C5	1.365 (4)	C20—C21	1.409 (3)
C4—H4	0.9300	C20—H20	0.9300
C5—C6	1.414 (3)	C21—C26	1.416 (3)
C5—H5	0.9300	C21—C22	1.421 (3)
C6—C7	1.411 (3)	C22—C23	1.365 (4)
C7—C8	1.358 (3)	C22—H22	0.9300
C7—H7	0.9300	C23—C24	1.403 (4)
C8—C9	1.418 (3)	C23—H23	0.9300
C8—H8	0.9300	C24—C25	1.369 (3)
C9—N1	1.325 (3)	C24—H24	0.9300
C9—C10	1.474 (3)	C25—O2	1.361 (3)
C10—N2	1.327 (3)	C25—C26	1.414 (3)
C10—N3	1.381 (3)	C26—N4	1.374 (3)
C11—N2	1.385 (3)	C27—C28	1.4463 (15)
C11—C12	1.400 (3)	C27—H27A	0.9600
C11—C16	1.402 (3)	C27—H27B	0.9600
C12—C13	1.378 (4)	C27—H27C	0.9600
C12—H12	0.9300	C28—C29	1.358 (6)
C13—C14	1.398 (4)	C28—C30^i^	1.365 (6)
C13—H13	0.9300	C29—C30	1.351 (9)
C14—C15	1.381 (3)	C29—H29	0.9300
C14—H14	0.9300	C30—C28^i^	1.365 (6)
C15—C16	1.394 (3)	C30—H30	0.9300
C15—H15	0.9300	O1—H1	0.8200
C16—N3	1.385 (3)	O2—H2	0.8200
C17—N3	1.462 (3)		
			
N1—C1—C6	123.4 (2)	C18—C17—H17B	108.7
N1—C1—C2	117.63 (19)	H17A—C17—H17B	107.6
C6—C1—C2	119.0 (2)	N4—C18—C19	122.82 (19)
O1—C2—C3	120.0 (2)	N4—C18—C17	115.06 (17)
O1—C2—C1	120.0 (2)	C19—C18—C17	122.09 (18)
C3—C2—C1	120.0 (2)	C20—C19—C18	118.8 (2)
C2—C3—C4	120.4 (2)	C20—C19—H19	120.6
C2—C3—H3	119.8	C18—C19—H19	120.6
C4—C3—H3	119.8	C19—C20—C21	121.0 (2)
C5—C4—C3	121.2 (2)	C19—C20—H20	119.5
C5—C4—H4	119.4	C21—C20—H20	119.5
C3—C4—H4	119.4	C20—C21—C26	115.94 (19)
C4—C5—C6	120.1 (2)	C20—C21—C22	124.9 (2)
C4—C5—H5	119.9	C26—C21—C22	119.2 (2)
C6—C5—H5	119.9	C23—C22—C21	119.8 (2)
C7—C6—C5	124.3 (2)	C23—C22—H22	120.1
C7—C6—C1	116.4 (2)	C21—C22—H22	120.1
C5—C6—C1	119.3 (2)	C22—C23—C24	121.2 (2)
C8—C7—C6	120.6 (2)	C22—C23—H23	119.4
C8—C7—H7	119.7	C24—C23—H23	119.4
C6—C7—H7	119.7	C25—C24—C23	120.2 (2)
C7—C8—C9	118.8 (2)	C25—C24—H24	119.9
C7—C8—H8	120.6	C23—C24—H24	119.9
C9—C8—H8	120.6	O2—C25—C24	120.1 (2)
N1—C9—C8	123.0 (2)	O2—C25—C26	119.5 (2)
N1—C9—C10	118.97 (18)	C24—C25—C26	120.3 (2)
C8—C9—C10	117.98 (18)	N4—C26—C25	117.61 (19)
N2—C10—N3	112.54 (18)	N4—C26—C21	123.14 (19)
N2—C10—C9	122.02 (18)	C25—C26—C21	119.2 (2)
N3—C10—C9	125.44 (17)	C28—C27—H27A	109.5
N2—C11—C12	129.9 (2)	C28—C27—H27B	109.5
N2—C11—C16	109.73 (17)	H27A—C27—H27B	109.5
C12—C11—C16	120.4 (2)	C28—C27—H27C	109.5
C13—C12—C11	117.5 (2)	H27A—C27—H27C	109.5
C13—C12—H12	121.2	H27B—C27—H27C	109.5
C11—C12—H12	121.2	C29—C28—C30^i^	121.6 (5)
C12—C13—C14	121.5 (2)	C29—C28—C27	117.7 (6)
C12—C13—H13	119.2	C30^i^—C28—C27	120.6 (6)
C14—C13—H13	119.2	C30—C29—C28	118.3 (5)
C15—C14—C13	121.9 (2)	C30—C29—H29	120.9
C15—C14—H14	119.0	C28—C29—H29	120.9
C13—C14—H14	119.0	C29—C30—C28^i^	120.1 (5)
C14—C15—C16	116.6 (2)	C29—C30—H30	120.0
C14—C15—H15	121.7	C28^i^—C30—H30	120.0
C16—C15—H15	121.7	C9—N1—C1	117.77 (18)
N3—C16—C15	132.0 (2)	C10—N2—C11	105.48 (17)
N3—C16—C11	106.02 (18)	C10—N3—C16	106.21 (16)
C15—C16—C11	122.00 (19)	C10—N3—C17	129.92 (17)
N3—C17—C18	114.36 (16)	C16—N3—C17	123.86 (16)
N3—C17—H17A	108.7	C18—N4—C26	118.16 (17)
C18—C17—H17A	108.7	C2—O1—H1	109.5
N3—C17—H17B	108.7	C25—O2—H2	109.5
			
N1—C1—C2—O1	2.7 (3)	C19—C20—C21—C22	179.3 (2)
C6—C1—C2—O1	−177.24 (19)	C20—C21—C22—C23	179.0 (2)
N1—C1—C2—C3	−178.68 (19)	C26—C21—C22—C23	−0.6 (3)
C6—C1—C2—C3	1.4 (3)	C21—C22—C23—C24	0.3 (4)
O1—C2—C3—C4	177.8 (2)	C22—C23—C24—C25	−0.1 (4)
C1—C2—C3—C4	−0.8 (3)	C23—C24—C25—O2	178.7 (2)
C2—C3—C4—C5	0.0 (4)	C23—C24—C25—C26	0.2 (4)
C3—C4—C5—C6	0.2 (4)	O2—C25—C26—N4	1.5 (3)
C4—C5—C6—C7	−178.4 (2)	C24—C25—C26—N4	179.9 (2)
C4—C5—C6—C1	0.4 (3)	O2—C25—C26—C21	−179.1 (2)
N1—C1—C6—C7	−2.3 (3)	C24—C25—C26—C21	−0.6 (3)
C2—C1—C6—C7	177.70 (19)	C20—C21—C26—N4	0.6 (3)
N1—C1—C6—C5	178.91 (19)	C22—C21—C26—N4	−179.78 (19)
C2—C1—C6—C5	−1.1 (3)	C20—C21—C26—C25	−178.89 (19)
C5—C6—C7—C8	−178.7 (2)	C22—C21—C26—C25	0.8 (3)
C1—C6—C7—C8	2.5 (3)	C30^i^—C28—C29—C30	−1.2 (8)
C6—C7—C8—C9	−0.7 (3)	C27—C28—C29—C30	−178.0 (5)
C7—C8—C9—N1	−1.8 (3)	C28—C29—C30—C28^i^	1.2 (8)
C7—C8—C9—C10	178.01 (19)	C8—C9—N1—C1	2.2 (3)
N1—C9—C10—N2	179.53 (18)	C10—C9—N1—C1	−177.69 (17)
C8—C9—C10—N2	−0.3 (3)	C6—C1—N1—C9	0.0 (3)
N1—C9—C10—N3	−0.2 (3)	C2—C1—N1—C9	180.00 (18)
C8—C9—C10—N3	179.92 (18)	N3—C10—N2—C11	−0.2 (2)
N2—C11—C12—C13	−179.8 (2)	C9—C10—N2—C11	−179.95 (17)
C16—C11—C12—C13	−0.5 (3)	C12—C11—N2—C10	178.8 (2)
C11—C12—C13—C14	−0.3 (3)	C16—C11—N2—C10	−0.6 (2)
C12—C13—C14—C15	0.6 (4)	N2—C10—N3—C16	0.8 (2)
C13—C14—C15—C16	−0.1 (4)	C9—C10—N3—C16	−179.41 (18)
C14—C15—C16—N3	178.5 (2)	N2—C10—N3—C17	−178.58 (18)
C14—C15—C16—C11	−0.8 (3)	C9—C10—N3—C17	1.2 (3)
N2—C11—C16—N3	1.0 (2)	C15—C16—N3—C10	179.6 (2)
C12—C11—C16—N3	−178.36 (18)	C11—C16—N3—C10	−1.1 (2)
N2—C11—C16—C15	−179.55 (19)	C15—C16—N3—C17	−1.0 (3)
C12—C11—C16—C15	1.1 (3)	C11—C16—N3—C17	178.35 (17)
N3—C17—C18—N4	158.92 (17)	C18—C17—N3—C10	−81.6 (3)
N3—C17—C18—C19	−23.0 (3)	C18—C17—N3—C16	99.1 (2)
N4—C18—C19—C20	2.3 (3)	C19—C18—N4—C26	−2.8 (3)
C17—C18—C19—C20	−175.59 (19)	C17—C18—N4—C26	175.22 (16)
C18—C19—C20—C21	−0.3 (3)	C25—C26—N4—C18	−179.18 (18)
C19—C20—C21—C26	−1.1 (3)	C21—C26—N4—C18	1.4 (3)

Symmetry codes: (i) −*x*+2, −*y*+2, −*z*+1.

### Hydrogen-bond geometry (Å, °)

**Table:**

*D*—H···*A*	*D*—H	H···*A*	*D*···*A*	*D*—H···*A*
O1—H1···N1	0.82	2.29	2.745 (3)	116
O1—H1···N4	0.82	2.47	3.131 (3)	139
O2—H2···N4	0.82	2.27	2.722 (3)	116
O2—H2···N2^ii^	0.82	2.55	3.145 (3)	131

Symmetry codes: (ii) *x*+1, *y*, *z*.
